# The role of ethics consultants in healthcare: a pillar of integrity and transparency in the health services. An experience in Lleida

**DOI:** 10.3389/frhs.2025.1558269

**Published:** 2025-06-12

**Authors:** Maria José Ruiz-Montilla, Oriol Yuguero

**Affiliations:** ^1^ Quality Department, University Hospital Arnau de Vilanova de Lleida, Lleida, Spain; ^2^ Faculty of Medicine, University of Lleida, Lleida, Spain; ^3^ Ehealth Center, Universitat Oberta de Catalunya, Barcelona, Spain

**Keywords:** bioethics, consultant, healthcare, quality of care, emergencies

## Abstract

Healthcare ethics has evolved since the Hippocratic Oath was adopted, to become a complex multifaceted discipline known as bioethics. This article aims to explore the role played by healthcare ethics consultants in depth, analysing how their intervention is crucial to ensuring transparency, integrity, and trust in health institutions. These consultants have proved very helpful in high-pressure healthcare areas such as emergency services and intensive care medicine, where rapid responses are often essential. The growing complexity of healthcare, driven by technological advances and economic pressure, makes the role of ethics consultants more critical than ever.

## Introduction

Healthcare ethics committees (HECs) have become essential structures within healthcare institutions, guiding clinical practice through the resolution of ethical dilemmas. Since their establishment in the 1970s in the United States (1) and later in Spain (2), HECs have played a crucial role in promoting transparency, patient rights, and responsible decision-making. Their tasks include advising on issues such as informed consent, end-of-life care, treatment refusal, and conflicts of interest (3).

In parallel, the figure of the healthcare ethics consultant has emerged as a key element in responding to urgent ethical challenges, particularly in high-pressure environments such as emergency departments and intensive care units (4). Ethics consultants provide real-time advice to healthcare professionals, helping them navigate complex decisions while respecting patient autonomy and institutional values.

Despite the recognized value of HECs and ethics consultants, significant barriers remain to their full integration within clinical practice, including limited accessibility, lack of ethical culture in certain institutions, and the tension between clinical pressures and ethical deliberation (5, 6).

## Context

In Spain, the implementation of healthcare ethics committees began in the early 1980s. One of the first in Catalonia was created at Arnau de Vilanova University Hospital (7). Building on these precedents, the region of Lleida has recently initiated a novel approach to strengthen ethics support in healthcare.

This article explores the dual role of healthcare ethics committees and ethics consultants, using the experience of Lleida as a case study. It analyzes the main bioethical challenges encountered, the barriers and facilitators faced in the implementation process, and how the local model compares to international experiences. It concludes by offering reflections on the lessons learned and recommendations for strengthening ethical practice within healthcare institutions.

In Spain, the development of healthcare ethics committees began with the pioneering efforts of individual hospitals. The first Healthcare Ethics Committee (HEC) was established in 1981 at the Sant Joan de Déu Hospital in Barcelona (2), under the leadership of Dr. Francesc Abel Fabre. Following this example, the Arnau de Vilanova University Hospital in Lleida created its own HEC in 1992, becoming the second in Catalonia (7). Initially focused on traditional ethical dilemmas, the committee progressively expanded its role to respond to the increasing complexity of clinical practice.

## Description of the programmatic elements

Recognizing the need for more immediate and accessible ethical support, in April 2019, the healthcare region of Lleida introduced the figure of the healthcare ethics consultant. **A group of five trained bioethics experts — including three physicians and two nurses —** began offering direct ethical consultation to healthcare professionals and patients. **Four of the five members hold a PhD in bioethics, each focusing on different aspects of clinical ethics, which highlights the depth and diversity of expertise within the group**. The system allowed 24/7 contact through a hospital-operated switchboard, ensuring privacy and rapid response.

The Lleida model shares similarities with leading international experiences, such as the ethics consultation services at New York Presbyterian-Weill Cornell Medical Center (4). However, it was specifically adapted to the regional needs: a mid-sized population, a centralized tertiary hospital, and limited access to real-time legal consultation services. These local adaptations have been key to the project's success and sustainability.

## Analysis of cases: linking bioethical challenges to real practice

Since the introduction of the healthcare ethics consultant service in Lleida, a significant number of consultations have been registered, particularly from emergency and intensive care units. An analysis of 35 cases attended between 2019 and mid-2024 reveals key trends reflecting the primary ethical concerns faced by healthcare professionals in high-pressure settings. Since 2019, the ethics consultants in Lleida have received many queries involving different types of dilemmas. Table 1 shows the number of cases the ethics consultants dealt with between 2019 and June 2024.

**Table 1:** Summary of ethics consultant cases by year and by matter.

Reasons for consultation	Year						Total
	2019	2020	2021	2022	2023	2024	
Refusal of treatment		2	2	2	2	2	10
Termination of pregnancy				1	1	1	3
Autonomy	1	1		5	1		8
Euthanasia					1		1
Information				1			1
Genetics and genetic diseases				1			1
Limitation of therapeutic effort		1	1	2	3	1	8
Others				2	1		3
Total	1	4	3	14	9	4	35

The two most frequent areas of consultation were treatment refusal (28.5%) and limitation of therapeutic effort (22.8%). These two categories highlight recurring ethical tensions between respecting patient autonomy and ensuring beneficence, especially in contexts where rapid decisions are crucial (3). For instance, treatment refusal cases often involved patients who, despite understanding the potential consequences, chose not to receive recommended life-saving interventions.

Cases related to **autonomy** (22.8%) also emerged as a significant category. Ethical dilemmas frequently involved disputes about decision-making capacity, informed consent, and respecting the preferences of vulnerable populations, including elderly or cognitively impaired patients (8). These cases emphasize the need for structured ethical support when clinical judgments intersect with legal and moral obligations.

Other categories, such as termination of pregnancy, information disclosure, genetic conditions, and euthanasia, though less frequent, point to the evolving bioethical landscape shaped by societal debates and legal reforms (9).

Interestingly, many of the cases managed by the ethics consultants could be addressed without requiring full deliberation by the Healthcare Ethics Committee (HEC). This demonstrates the value of immediate, flexible ethical advice provided by consultants, a model consistent with international experiences at institutions like the Cleveland Clinic and Boston Children's Hospital (10).

Overall, the pattern of consultations underscores the critical role of ethics consultants in guiding clinical teams through morally complex situations, improving the ethical quality of healthcare delivery, and fostering an institutional culture of ethical sensitivity and reflection.

## Discussion

The implementation of healthcare ethics consultation services has evolved differently across institutions, adapting to the needs and resources of each healthcare system. Leading centers such as the Mayo Clinic, the Cleveland Clinic, and New York-Presbyterian Weill Cornell Medical Center have pioneered structured models of clinical ethics consultation that serve as important references for newer initiatives like the one developed in Lleida.

At the Mayo Clinic, the Ethics Consultation Service integrates seamlessly with the clinical workflow, offering non-binding recommendations aimed at supporting, rather than dictating, clinical decisions (11). Consultants focus on enhancing communication among patients, families, and healthcare teams, a model that has been associated with improved satisfaction and conflict resolution rates.

The Cleveland Clinic's approach emphasizes proactive ethics consultation, often involving early intervention in complex cases before conflicts escalate (12). Their service is distinguished by systematic training programs for consultants and by formal processes for tracking consultation outcomes, promoting quality improvement and institutional learning.

At Cornell, the Ethics Consultation Service places strong emphasis on responsiveness and accessibility, operating through a decentralized model where ethics consultants are embedded within clinical departments (13). This structure fosters a culture of ethical awareness and immediate support, enabling clinicians to address ethical concerns at the bedside.

The Lleida experience shares fundamental principles with these established models, particularly the focus on timely, accessible, and educationally oriented ethics consultations. However, important adaptations were necessary to meet local needs: the service operates within a single tertiary hospital rather than a network of institutions, resources are more limited, and there is greater reliance on voluntary participation of consultants. Despite these differences, the Lleida model successfully integrates core elements of international best practices while maintaining flexibility to address the specific ethical challenges of its healthcare environment.

## Conclusions

The development and implementation of a healthcare ethics consultant service at Arnau de Vilanova University Hospital in Lleida represents a significant step forward in promoting ethical reflection within clinical practice. By building upon the foundations established by the hospital's longstanding Healthcare Ethics Committee, the initiative demonstrated that is possible to create an accessible and effective ethics consultation model. The focus on real-time, bedside ethical support has proven especially valuable in high-pressure units such as emergency services and intensive care.

Although inspired by leading international models, the Lleida experience was carefully adapted to local circumstances, balancing innovation with pragmatism. Future efforts should aim to strengthen the service through systematic training programs, structured evaluation of consultation outcomes, and broader dissemination of the ethics consultant model across other hospitals in the region.

In conclusion, the Lleida experience illustrates how ethics consultation can be successfully embedded in clinical practice, even outside of large academic centers, and highlights the importance of flexible, context-sensitive approaches to institutional bioethics.

## Data Availability

The original contributions presented in the study are included in the article/Supplementary Material, further inquiries can be directed to the corresponding author.
